# Editorial: Natural animal models of diseases

**DOI:** 10.3389/fvets.2022.1056317

**Published:** 2022-11-17

**Authors:** Fernando Capela e Silva, João R. Mesquita, Maria Anjos Pires, Alberto Muñoz-Prieto

**Affiliations:** ^1^Department of Medical and Health Sciences, School of Health and Human Development, MED-Mediterranean Institute for Agriculture, Environment and Development, University of Évora, Évora, Portugal; ^2^ICBAS-Institute for the Biomedical Sciences Abel Salazar, School of Medicine and Biomedical Sciences, University of Porto, Porto, Portugal; ^3^CECAV-Animal and Veterinary Research Center, School of Agricultural and Veterinary Sciences, University of Trás-os-Montes and Alto Douro, Vila Real, Portugal; ^4^Faculty of Veterinary Medicine, University of Zagreb, Zagreb, Croatia

**Keywords:** natural animal models, spontaneous animal models, comparative models, translational medicine, One Health

The use of animals in research has been the subject of several discussions due to the large number needed and the suffering caused, existing a worldwide trend to reevaluate their use in experimentation and which culminated in the program called 3Rs (Reduction, Refinement, and Replacement). This concept is based on reducing the number of animals used in research, minimizing its pain and discomfort, and replacing the *in vivo* tests. Several alternative methodologies have been implemented and validated and in addition to *in vitro* tests, some spontaneous models have been used to understand the pathological mechanisms of diseases, since some of them in humans are similar to those that occur in some domestic species, such as dogs, cats, horses, and others. These species share the same environments and are exposed to the same contaminants, their biology is similar, and they respond in a similar way from a physiological, pathological, and therapeutic point of view.

As an example, some types of cancer (like the ocular squamous cell carcinoma, breast/mammary tumors, and melanomas) affect humans and several of these species; and, dogs and cats, develop some pathways of human metabolic syndrome (as obesity, insulin resistance, increased blood pressure, and hyperlipidemia) and other similar diseases such as inflammatory bowel disease, mitral valve disease, epilepsy, chronic kidney disease, etc.

In this way, these “*Natural animal models of diseases*” are a very interesting and promising tool, both from an ethical point of view, avoiding the use of experimental laboratory models, and from a biological point of view: allowing to prevent diseases in these animals, and serving as a models of the same conditions in humans, to understand the pathophysiological mechanisms, with an impact on the prevention and progression of diseases, and in the search for new strategies and therapeutic approaches. On the other hand, this way of acting helps to emphasize and consolidate the One Health approach.

In this Research Topic of Frontiers in Veterinary Science/Comparative and Clinical Medicine, four manuscripts were published: Two Review and two Original Research Articles, whose main contributions and results are briefly presented below.

Epilepsy is a common neurological disease in humans and domestic dogs and cats, characterized by an enduring predisposition to generate spontaneous recurrent epileptic seizures, making dogs an ideal translational model for its study (Löscher). In both species, epilepsy is a group of disorders characterized by a wide range of clinical signs, age of onset, and underlying causes, and as in humans, status epilepticus is one of the most common neurological emergencies in dogs with epilepsy. In the manuscript *Dogs as a natural animal model of epilepsy*, Löscher makes an exhaustive and very detailed characterization of epilepsy in dogs, namely its pathogeny, its treatment, the experimentally induced seizures in non-epileptic dogs, and the use of dogs as a model of birth asphyxia and neonatal seizures and as a translational model in epilepsy research and antiseizure drug development. According to the author, the study of epilepsy in dogs is naturally important in veterinary medicine, being also a very interesting spontaneous model in the study of this condition in human medicine. Moreover, since resistance to antiseizure medications is considered to be an important issue in both canine and human epilepsy, dogs can also be used as models to evaluate drug resistance mechanisms and develop novel therapeutic approaches.

Many outbreaks of foodborne illness, typically associated with the consumption of undercooked and processed foods, derive from contamination with bovine feces containing enterohemorrhagic *Escherichia coli* serotype O157:H7. Many of the factors that regulate enteric colonization by *E. coli* O157:H7 in cattle and how they respond to the bacteria are unknown, namely intestinal colonization sites, patterns of excretion, interactions with the enteric microbiota and host immune responses to infection (Lange et al.). Thus, there are currently no effective mitigations for this situation. The logistical issues associated with bovine production, the cost and the marked genetic, physiological and microbial heterogeneity have hampered the ability to clarify the main aspects of the host-pathogen-microbiota interaction. Although mice are not widely used as a model for livestock species, its use has been effective in assessing gut colonization, host responses to infection, and in determining how the physiological state of the host (e.g., due to physiological stress) and the enteric microbiota influence colonization locations and the disease (Lange et al.). In the manuscript *Enteric Escherichia coli O157:H7 in Cattle, and the use of mice as a model to elucidate key aspects of the host-pathogen-microbiota interaction: A review*, Lange et al. review the literature considered relevant on colonization and pathogenesis, including the gaps that currently exist, providing information on how murine models can be used to elucidate mechanisms for developing mitigations for *E. coli* O157:H7 infection in cattle.

The diagnosis of acute pulmonary thromboembolism in dogs is a challenging issue in veterinary medicine, but changes in echocardiographic indices are not well-documented (Morita et al.). Using beagles as a model, the work by Morita et al., *Evaluation of right ventricular function and dyssynchrony in a dog model of acute pulmonary embolism: Diagnostic utility and reversibility*, aimed to validate the relationship between echocardiographic indices and cardiac catheterization variables in dogs with acute pulmonary embolism and to identify a useful echocardiographic index for its diagnosis. Acute pulmonary embolism was produced by injection of microspheres, and echocardiographic indices were measured, including right ventricular (RV) Tei index (myocardial performance index), RV longitudinal strain, and dyssynchrony index by speckle tracking echocardiography, transmitral flow, and eccentricity index. The results obtained suggest that the RV Tei index is a useful echocardiographic index for the diagnosis of acute pulmonary embolism and that ventricular interdependence may be an important factor causing low cardiac output in dogs in this situation.

Excessive exposure to adrenocortical hormones can cause steroid-induced hepatopathy, a liver disease, a condition that can progress to hepatocellular carcinoma (HCC), one of the most common primary liver tumors in humans and also described in dogs. The concomitance of hyperadrenocorticism (HAC) in dogs with HCC raised the hypothesis that adrenal steroid alterations may be involved in hepatocarcinogenesis, a situation analyzed by Oo et al. in the research *Serum steroid profiling of hepatocellular carcinoma associated with hyperadrenocorticism in dogs: A preliminary study*. By using liquid chromatography together with tandem mass spectrometry (LC/MS/MS), 19 steroids (14 steroids and five metabolites) were quantitatively measured in the basal serum of 15 dogs with HCC, 15 with HAC and 10 with both diseases. The results obtained allowed to conclude that the developed LC/MS/MS was useful for measuring steroid hormones and that although it was evident that HAC was concomitant in dogs with HCC, even though none of the serum steroids was suggested to be involved in HCC development.

## Author contributions

FC wrote the draft which was amended and revised by JM, MP, and AM-P. All authors contributed to the article and approved the submitted version.

## Conflict of interest

The authors declare that the research was conducted in the absence of any commercial or financial relationships that could be construed as a potential conflict of interest.

## Publisher's note

All claims expressed in this article are solely those of the authors and do not necessarily represent those of their affiliated organizations, or those of the publisher, the editors and the reviewers. Any product that may be evaluated in this article, or claim that may be made by its manufacturer, is not guaranteed or endorsed by the publisher.

